# Combinations of intrinsic capacity and frailty and their associations with self-rated health in community-dwelling older adults

**DOI:** 10.1007/s40520-025-03189-z

**Published:** 2025-09-06

**Authors:** Daijo Shiratsuchi, Hyuma Makizako, Shoma Akaida, Yuto Miyake, Ryoji Kiyama, Takayuki Tabira, Mana Tateishi, Rei Otsuka, Toshihiro Takenaka, Takuro Kubozono, Mitsuru Ohishi

**Affiliations:** 1 Department of Physical Therapy, School of Health Sciences, Faculty of Medicine, Kagoshima University, 8-35-1 Sakuragaoka, Kagoshima, 890- 8544 Japan; 2 Faculty of Medicine, Kagoshima University, Kagoshima, Japan; 3 Department of Preventive Gerontology, Center for Gerontology and Social Science, Research Institute, National Center for Geriatrics and Gerontology, Obu, Japan; 4 Graduate School of Health Sciences, Kagoshima University, Kagoshima, Japan; 5 Department of Occupational Therapy, School of Health Sciences, Faculty of Medicine, Kagoshima University, Kagoshima, Japan; 6 Department of Epidemiology of Aging, National Center for Geriatrics and Gerontology, Obu, Japan; 7 Tarumizu Municipal Medical Center Tarumizu Chuo Hospital, Kagoshima, Japan; 8 Department of Cardiovascular Medicine and Hypertension, Graduate School of Medical and Dental Sciences, Kagoshima University, Kagoshima, Japan

**Keywords:** Self-rated health, Healthy aging, Geriatric assessment, Community-based study, Older adults

## Abstract

**Background:**

Intrinsic capacity (IC) and frailty are distinct but complementary frameworks for understanding the heterogeneity of aging. Although both have been linked to self-rated health, little is known about how their combined status relates to older adults’ health perceptions. This cross-sectional study investigated how combinations of IC and frailty status were associated with self-rated health among community-dwelling older adults.

**Method:**

Data from 593 participants (mean age: 73.9 years, female: 62.2%) in the 2019 Tarumizu Study were analyzed. IC was evaluated across five key screening domains (cognition, vitality, sensory, locomotion, and psychological) using a 10-point scale, with scores ≥ 9 classified as high IC. Frailty was assessed using the Japanese version of the Cardiovascular Health Study criteria and dichotomized into robustness and frailty (pre-frailty or frailty). Self-rated health was assessed with a four-point item and dichotomized into good or poor. Participants were classified into four groups: robust with high IC, frailty with high IC, robust with low IC, and frailty with low-IC.

**Results:**

Group proportions were: 30.0% (robust with high IC), 23.1% (frailty with high IC), 15.9% (robust with low IC), and 31.0% (frailty with low IC). Multivariable logistic regression showed that only the frailty with low-IC group had significantly greater odds of poor self-rated health compared to the robust with high-IC group (OR = 3.55, 95% CI: 1.34–9.36).

**Conclusions:**

Co-occurrence of low IC and frailty was significantly associated with poor self-rated health. These findings suggest that considering IC and frailty may enhance understanding of self-rated health in later life.

## Introduction

Self-rated health is a widely used indicator of overall health status, particularly in older adults, and has been shown to predict various adverse outcomes, including functional decline, chronic disease, and mortality [1, 2]. As a self-perceived assessment of physical, mental, and social well-being, self-rated health captures dimensions of health that may not be fully reflected in objective clinical measures [3]. Given its simplicity, validity, and predictive value, self-rated health has become an essential tool in both public health surveillance and aging research.

Among the various age-related health indicators, frailty and intrinsic capacity (IC) have received increasing attention as complementary frameworks to understand the heterogeneity of aging. Frailty is a clinically recognized condition characterized by increased vulnerability to stressors due to the decline of multiple physiological systems, and has been associated with adverse outcomes such as falls, hospitalization, and mortality [4]. In contrast, IC, as proposed by the World Health Organization (WHO), emphasizes the preservation of physical and mental capacities that contribute to functional ability in later life [5, 6]. Although conceptually distinct, IC and frailty describe related dimensions of aging. IC reflects capacity, whereas frailty indicates deficit. Frailty may arise when IC falls below a critical threshold. However, IC is not simply the opposite of frailty. The two differ in orientation (capacity-based vs. deficit-based), timing (proactive vs. reactive), and purpose (promotion vs. risk screening) [7]. When used together, they offer a more complete picture: frailty identifies vulnerability, and IC highlights remaining strength that may support resilience. This complementarity has important implications for care planning and healthy aging promotion.

Although both IC and frailty have been individually associated with various objective and self-rated health outcomes, few studies have examined how their combination is related to self-rated health in older adults [8]. A recent scoping review of IC-related outcomes reported only one study that examined self-rated health and no studies that simultaneously examined IC and frailty in relation to self-rated health [8, 9]. To deepen our understanding of older adults’ health, it is important to consider both biomedical risk factors and self-rated health perceptions. Incorporating both perspectives enables a more comprehensive evaluation of health status and offers valuable insights for the development of appropriate interventions and support strategies.

Therefore, in this study, we aimed to investigate how IC and frailty status are associated with self-rated health in community-dwelling older adults. Participants were categorized into four groups based on their IC and frailty status: robust with high IC, frail with high IC, robust with low IC, and frail with low IC. Group differences in self-rated health were analyzed using data from a community-based health survey. By simultaneously considering IC and frailty, we aimed to provide a more nuanced understanding of how older adults perceive their overall health, which may not be fully captured by either concept alone. We expected to observe that, even in the presence of frailty, individuals with high IC would be less likely to report poor self-rated health.

## Methods

### Study design and participants

This cross-sectional study used data from the Tarumizu Study, a community-based health survey conducted between June and December 2019 in Tarumizu City, Kagoshima Prefecture, Japan. The Tarumizu Study is an ongoing community-based research project aimed at assessing the health status and associated factors among community-dwelling older adults [10]. In 2019, Tarumizu city had a population of 14,754 individuals, with 41.7% (6,149 individuals) aged 65 years or older. Participants were recruited through multiple public outreach methods, including the official municipal website, telephone and in-person registration at local offices, postcard applications, and announcements in the city’s public information magazine. A total of 687 community-dwelling older adults aged 65 years and older participated in the 2019 survey. Participants were excluded if they had a certified need for long-term care (*n* = 14), disability in basic activities of daily living (*n* = 12), history of stroke (*n* = 32), dementia (*n* = 6), or missing data (*n* = 30). The certified need for long-term care was verified using municipal records obtained from Tarumizu city. ADL disability was determined based on self-reporting. History of stroke and dementia were assessed through structured interviews conducted by trained physicians or nurses. After applying these criteria, 593 participants were included in the final analysis (mean age: 73.9 ± 6.3 years; 62.2% female). All participants provided written informed consent prior to enrollment. The study was approved by the Ethics Committee on Epidemiological and Related Studies, Sakuragaoka Campus, Kagoshima University (approval number: 170351), and was conducted in accordance with the principles of the Declaration of Helsinki.

### Assessment of IC

IC was assessed based on the concept proposed by the WHO and the scoring method developed by López-Ortiz et al. [11, 12]. Five domains, including cognition, locomotor capacity, vitality, sensory function (vision and hearing), and psychological capacity, were selected in line with previous studies and expert discussions, considering the available cohort data. The assessment items for each domain were selected based on the screening components described in the ICOPE Handbook to ensure conceptual alignment with the WHO framework. Each domain includes two items scored as 1 (no impairment) or 0 (impairment), resulting in domain scores ranging from 0 to 2 and a total IC score ranging from 0 to 10.

Cognition was assessed using two items derived from the Kihon Checklist and Mini-Cog [13, 14]. Participants were asked, “Do you find yourself not knowing today’s date?” (Kihon Checklist), and were administered a 3-item recall task (Mini-Cog).

Vitality was evaluated using body mass index (BMI) and appetite. A low BMI was defined according to the GLIM criteria ​ [15] as < 18.5 kg/m² for those younger than 70 years and < 20.0 kg/m² for those aged 70 years or older. Appetite was assessed using the first item of the Simplified Nutritional Appetite Questionnaire [16], with responses of “poor” or “very poor” (on a 4-point scale excluding “average”) indicating impairment.

Sensory function was assessed based on self-reported vision and hearing. For vision, participants were scored as impaired if they used ophthalmic medications. For hearing, they were asked, “Do you feel disturbance in the conversation with the other person (in a one-on-one setting)?”, with responses of “slightly audible” or “disturbed” indicating impairment [17].

Locomotor capacity was assessed using the five-time chair stand test and usual gait speed. According to AWGS 2019 criteria [18], impairment was defined as a chair stand time ≥ 12 s and gait speed < 1.0 m/s.

Psychological capacity was assessed using two items related to depressive symptoms [19, 20]: “Have you dropped many of your activities and interests?” and “Do you feel happy most of the time?”, scored as impaired if the participant answered “yes” to the former or “no” to the latter.

Participants were categorized into two groups based on the total IC score (range: 0–10): high IC (scores ≥ 9) and low IC (scores ≤ 8), following the classification method proposed by López-Ortiz et al. [12].

### Assessment of frailty

Frailty was assessed using the revised Japanese version of the Cardiovascular Health Study (revised J-CHS) criteria, which was updated based on expert consensus by the Japanese Association on Sarcopenia and Frailty (JASF), as reported by Satake and Arai [21]. This tool consists of five components: (1) unintentional weight loss (≥ 2 kg in the past 6 months), (2) weakness (grip strength < 28 kg for men or < 18 kg for women), (3) exhaustion (feeling tired without reason in the past 2 weeks), (4) slowness (gait speed < 1.0 m/s), and (5) low physical activity (reporting no engagement in moderate or low levels of physical exercise for health). Grip strength was measured once in the standing position using a handheld dynamometer (Grip-D; Takei Ltd., Niigata, Japan). The maximum value of the dominant hand was used. Gait speed was assessed by conducting a standardized 10-m walk test along a 14-m walkway, which included 2 m for acceleration, 10 m for measurement, and 2 m for deceleration. Trained physiotherapists instructed the participants to walk at their usual pace. Gait speed was measured using a validated infrared gait analysis system (YW; Yagami Inc., Aichi, Japan) that has been previously employed in studies with this cohort [22, 23]. Each component was scored as 1 if present and 0 if absent, resulting in a total score ranging from 0 to 5. Participants were categorized as frail (score 3–5), pre-frail (score 1–2), or robust (score 0), in accordance with the revised J-CHS criteria. For this study, frailty status was dichotomized into robust and frail groups [24], with the latter including both pre-frail and frail individuals for all analyses.

### Combination of IC and frailty

Participants were categorized into four mutually exclusive groups based on their intrinsic capacity (IC: high/low) and frailty status (robust/frail): (1) robust with high-IC, (2) frail with high-IC, (3) robust with low-IC, and (4) frail with low-IC. These groups represent, respectively: no impairment (reference group), frailty alone, low IC alone, and both conditions combined.

### Assessment of self-rated health

Self-rated health was assessed using a single-item question: “Would you normally consider yourself healthy?” with four response options: “very healthy,” “fairly healthy,” “fairly unhealthy,” and “very unhealthy” ​ [25]. In line with previous studies, responses were dichotomized into good self-rated health (“very healthy” or “fairly healthy”) and poor self-rated health (“fairly unhealthy” or “very unhealthy”).

### Other variables

The following variables were collected to characterize the participants’ health status and were used for descriptive purposes or as covariates in the multivariate analysis. Demographic and lifestyle characteristics included age (continuous), sex (male or female), years of education (continuous), and living arrangements (living alone or not). Clinical characteristics included BMI (kg/m²), number of regularly used medications (continuous), and presence of chronic conditions. The chronic diseases assessed in this study included hypertension, hyperlipidemia, diabetes mellitus, osteoarthritis, respiratory diseases, and cancer. All information was obtained through self-administered questionnaires, interviews with physicians or nurses, and physical assessments performed as part of a community-based health survey.

### Statistical analysis

Descriptive statistics were calculated for all the variables. Continuous variables are expressed as means and standard deviations, while categorical variables are presented as frequencies and percentages. Group comparisons were performed using one-way analysis of variance (ANOVA) for continuous variables and chi-square tests for categorical variables. Bonferroni correction was applied in all post-hoc analyses to account for multiple comparisons among the four groups. To examine the associations between IC, frailty, and self-rated health, we conducted two sets of logistic regression analyses. First, we assessed the independent associations of low IC and frailty with poor self-rated health using separate models, each adjusted for age, sex, years of education, number of medications, and living arrangements. Second, we evaluated the combined effect of IC and frailty by categorizing participants into four groups (robust with high IC, frailty with high IC, robust with low IC, and frailty with low IC), using the robust with high-IC group as a reference. For the main analysis, we constructed both a crude model and an adjusted model that included the same covariates. These variables were selected based on their theoretical relevance and as per previous studies that frequently examined their associations with self-rated health in older adults [25, 26]. Odds ratios (OR) and 95% confidence intervals (CI) were determined. All analyses were conducted using IBM SPSS Statistics (version 30.0; IBM Japan, Tokyo, Japan), and a two-sided *P*-value < 0.05 was considered statistically significant.

## Results

### Participant characteristics

A total of 593 community-dwelling older adults (mean age: 73.9 ± 6.3 years; 62.2% female) were included in the analysis and classified into four groups based on their IC and frailty status: robust with high IC (*n* = 178, 30.0%), frail with high IC (*n* = 137, 23.1%), robust with low IC (*n* = 94, 15.9%), and frail with low IC (*n* = 184, 31.0%).

The frailty with low-IC group was the oldest (76.7 ± 7.0 years) and had the lowest level of educational attainment (10.9 ± 2.4 years), both of which were significantly different from those in the other groups (*P* < 0.001, Bonferroni-corrected post hoc comparisons). Details of participant characteristics are presented in Table 1.

**Table 1:** Participant characteristics stratified by the combination of intrinsic capacity and frailty status

Variables	Overall (*n* = 593)	Robust with high-IC group^a^ (*n* = 178)	Frailty with high-IC group^b^ (*n* = 137)	Robust with low-IC group^c^ (*n* = 94)	Frailty with low-IC group^d^ (*n* = 184)	*P* value	Post hoc
Age, years (mean ± SD)	73.9 ± 6.3	72.2 ± 5.3	73.1 ± 6.0	73.0 ± 5.2	76.7 ± 7.0	< 0.001	a, b, c < d
Sex, Female, n (%)	369 (62.2)	123 (69.1)	79 (57.7)	61 (64.9)	106 (57.6)	0.080	
Education, years (mean ± SD)	11.5 ± 2.2	11.8 ± 2.0	11.8 ± 2.4	11.8 ± 1.9	10.9 ± 2.4	< 0.001	a, b, c > d
Medications, number (mean ± SD)	3.1 ± 3.0	2.2 ± 2.6	3.5 ± 3.1	2.6 ± 2.5	3.9 ± 3.3	< 0.001	a < b, d; c < d
Living alone, n (%)	168 (28.3)	53 (29.8)	35 (25.5)	23 (24.5)	57 (31.0)	0.566	
BMI (mean ± SD)	23.1 ± 3.2	23.3 ± 2.9	23.7 ± 3.1	22.7 ± 3.0	22.7 ± 3.5	0.021	b > d
Chronic diseases, n (%)							
Hypertension	286 (48.2)	79 (44.4)	72 (52.6)	39 (41.5)	96 (52.2)	0.176	
Hyperlipidemia	169 (28.5)	48 (27.0)	46 (33.6)	29 (30.9)	46 (25.0)	0.348	
Diabetes mellitus	77 (13.0)	14 (7.9)	24 (17.5)	9 (9.6)	30 (16.3)	0.025	
Osteoarthritis	17 (2.9)	6 (3.4)	5 (3.6)	2 (2.1)	4 (2.2)	0.810	
Respiratory diseases	57 (9.6)	13 (7.3)	15 (10.9)	7 (7.4)	22 (12.0)	0.385	
Cancer	70 (11.8)	26 (14.6)	12 (8.8)	10 (10.6)	22 (12.0)	0.442	

Abbreviations: SD: standard deviation, BMI: body mass index. a–d: superscripts indicate results of post hoc comparisons. *P*-values were calculated using one-way analysis of variance (ANOVA) or chi-square tests, and bonferroni correction was applied to adjust for multiple comparisons in both tests, as appropriate

### Prevalence of poor self-rated health across groups

**Fig. 1:**
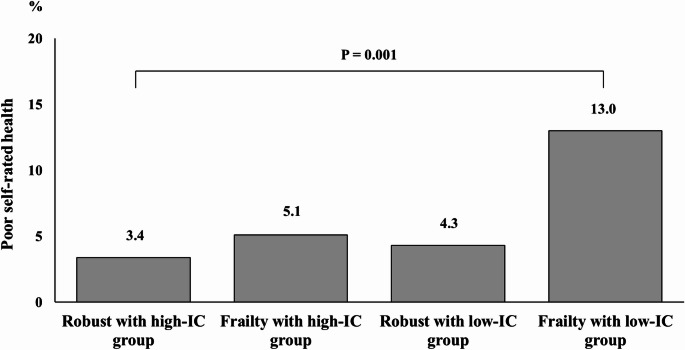
Proportion of participants with poor self-rated health across groups classified by intrinsic capacity and frailty status. Note: *n* = 178 robust with high IC, 137 frail with high IC, 94 robust with low IC, and 184 frail with low IC

The overall prevalence of poor self-rated health among participants was 6.9% (*n* = 41). Figure 1 illustrates the proportion of participants with poor self-rated health in each group. The prevalence was highest in the frailty with low-IC group (13.0%), followed by the high-IC group (5.1%), low-IC group (4.3%), and the robust with high-IC group (3.4%). Post hoc comparisons indicated that the prevalence was significantly higher in the frail with low IC group than in the robust with high IC group (*P* = 0.001).

### Associations of intrinsic capacity and frailty with poor self-rated health

To clarify the individual associations of IC and frailty with poor self-rated health, we first conducted separate logistic regression analyses (Table 2). After adjusting for age, sex, years of education, number of medications, and living arrangements, both low IC (OR: 2.32; 95% CI, 1.15–4.7, *P* = 0.019) and frailty (OR: 2.25; 95% CI, 1.05–4.83, *P* = 0.038) were significantly associated with poor self-rated health.

**Table 2:** Associations of intrinsic capacity and frailty with poor self-rated health

Independent variable	Model 1			Model 2	
	OR (95% CI)	*P*-value		OR (95% CI)	*P*-value
Intrinsic Capacity					
Low-IC group (vs. High-IC group)	2.32 (1.15–4.70)	0.019			
Frailty					
Frailty group (vs. Robust group)				2.25 (1.05–4.83)	0.038

Abbreviations: CI: Confidence interval, OR: Odds ratio

Adjusted for age, sex, education, number of medications, and living alone

### Associations between IC–frailty group and poor Self-rated health

We then examined the combined association of IC and frailty with poor self-rated health (Table 3). In the adjusted model, the frailty with low-IC group had significantly greater odds of reporting poor self-rated health compared to the robust with high-IC group (OR: 3.55, 95% CI: 1.34–9.36, *P* = 0.011). No significant associations were found for frailty in the high-IC group or for robustness in the low-IC group.

**Table 3:** Associations between intrinsic capacity–frailty group and poor self-rated health

Independent variable	Crude			Adjusted model	
	OR (95% CI)	*P*-value		OR (95% CI)	*P*-value
Robust with high-IC group	Reference			Reference	
Frailty with high-IC group	1.54 (0.51–4.70)	0.445		1.29 (0.42–4.01)	0.657
Robust with low-IC group	1.27 (0.35–4.63)	0.713		1.22 (0.33–4.45)	0.767
Frailty with low-IC group	4.30 (1.71–10.79)	0.002		3.55 (1.34–9.36)	0.011

Abbreviations: CI: Confidence interval, OR: Odds ratio

Adjusted for age, sex, education, number of medications, and living alone

## Discussion

In this study, we investigated how combinations of IC and frailty status are associated with self-rated health among community-dwelling older adults. We first examined the individual associations of low IC and frailty with poor SRH, which showed that both conditions were independently associated with poorer health perception, even after adjusting for relevant covariates. These findings highlighted the importance of considering both constructs when assessing subjective health in older populations. We then examined the combined effects of IC and frailty by classifying participants into four groups. The results revealed that only the frailty with low-IC group showed a significantly higher odds of reporting poor self-rated health, compared to the robust with high-IC group. In contrast, neither frailty in the high IC group nor robustness in the low IC group showed significant associations with poor self-rated health. These findings suggest that poorer self-rated health is more commonly observed among individuals with both low IC and frailty status. Although high IC was not statistically associated with better self-rated health among frail individuals, these findings indicate the potential complementary roles of IC and frailty in relation to self-rated health. Notably, individuals in the frailty with low-IC group were older, had fewer years of education, and were using more medications than those in the other groups. These characteristics have been associated with poor self-rated health in previous studies [2, 25] and might partially explain the higher prevalence of poor self-rated health observed in this group. These results support the importance of evaluating deficit-based indicators such as frailty, and capacity-oriented constructs such as IC when assessing health perceptions in older populations.

The prevalence of poor self-rated health in our cohort (6.9%) was substantially lower than rates reported in comparable international studies [27, 28]. This difference may primarily reflect variations in operational definitions: whereas many studies categorize “fair” or “moderate” responses as poor SRH>, we employed a stricter threshold limited to “fairly unhealthy” or “very unhealthy” responses. This more conservative approach aligns with findings from Japanese community-based studies using similar criteria [29], which consistently report lower prevalence rates. These systematic differences highlight how measurement methodology can significantly affect SRH prevalence estimates across studies.

Although frailty and IC represent contrasting frameworks for understanding aging, both have been individually associated with a wide range of adverse health outcomes, including disability, hospitalization, and mortality [30–32]. Frailty, which reflects accumulated deficits and physiological vulnerability, has been consistently linked to poor self-rated health in older populations [33]. Conversely, IC, which emphasizes physical and mental reserves, has shown positive associations with health-related quality of life and other functional outcomes [8]. Previous studies have generally considered these constructs separately. Frailty may emerge when IC deteriorates below a critical threshold; however, IC is not simply the inverse of frailty. The two differ in orientation: frailty captures vulnerability through accumulated deficits, whereas IC reflects remaining functional capacities. In this study, we assessed frailty using the revised J-CHS criteria, which reflect the physical phenotype of frailty. In contrast, IC encompasses broader domains, such as cognition, psychological function, and sensory ability. This distinction underscores the complementary nature of the two constructs. Frailty is often applied for risk identification and care prioritization, whereas IC is more applicable to prevention and health promotion strategies [7]. Our findings support the complementary roles of these constructs. In separate regression models, both low IC and frailty were independently associated with poor self-rated health. However, in the combined IC–frailty analysis, only the group exhibiting both low IC and frailty demonstrated a statistically significant association with poor self-rated health. This supports the notion that a combined evaluation of both capacity (IC) and vulnerability (frailty) offers a more comprehensive approach to understanding health heterogeneity in aging populations [7].

In clinical and community settings, assessments incorporating both frailty and IC may provide a more comprehensive understanding of how older adults perceive their health. Importantly, SRH may reflect not only physical vulnerability but also residual capacities and psychosocial adaptation. In this context, SRH can be considered as a subjective synthesis that integrates both deficit-oriented and capacity-oriented perspectives. Although frailty screening remains essential for identifying individuals at risk for adverse outcomes [34], integrating IC evaluation could help identify those who, despite physical vulnerability, maintain positive perceptions of health and functional reserves. Supporting the maintenance and enhancement of these residual IC domains may help sustain older adults’ overall well-being and independence. Moreover, promoting positive health assets, such as IC, rather than focusing solely on the avoidance of negative outcomes, aligns with current perspectives on healthy aging [35]. These findings suggest that clinical assessment of both vulnerability and preserved capacity could inform decisions regarding patient monitoring, follow-up scheduling, or specialist referral. This study has several limitations that should be considered when interpreting the findings. First, the cross-sectional design precluded any inference of causal relationships among IC, frailty, and self-rated health. Second, although our operational definition of IC was based on established domains and aligned with the WHO framework, it did not employ standardized Integrated Care for Older People (ICOPE) assessment tools. This methodological decision was guided by the structure of available data in community-based settings, but may limit the comparability of our findings with studies using the official ICOPE protocol or other standardized instruments. Third, some proxies, such as defining vision impairment based on the use of ophthalmic medications, may not fully capture domain-specific functional limitation and should be interpreted with caution. Fourth, usual gait speed (< 1 m/s) was used in the assessment of both IC (locomotor domain) and frailty. Although gait speed is a widely used indicator in both frameworks, this overlap might introduce some conceptual redundancy and should be taken into account when interpreting the results. Fifth, although we collected information on the major chronic diseases, we did not include the individual conditions as covariates in the main regression model to avoid statistical overfitting. Instead, we used the number of medications as a proxy for disease burden. Finally, the study sample consisted of community-dwelling older adults from a single rural city in Japan, who voluntarily participated in the health survey. This may limit the generalizability of our results and partly explains the relatively low prevalence of poor self-rated health observed in our findings.

## Conclusion

In this study, we examined the associations between combinations of IC, frailty status, and self-rated health among community-dwelling older adults. Although both low IC and frailty were independently associated with poor self-rated health in separate models, their co-occurrence demonstrated the strongest association in the combined analysis. These results suggest that considering IC and frailty may provide a more comprehensive understanding of how older adults perceive their health.

## Data Availability

The data used in this study cannot be publicly shared due to privacy concerns and ethical restrictions. The data can be obtained from the corresponding authors upon reasonable request.
